# Large-scale brainstem neuroimaging and genetic analyses provide new insights into the neuronal mechanisms of hypertension

**DOI:** 10.1016/j.xhgg.2024.100392

**Published:** 2024-12-10

**Authors:** Tiril P. Gurholt, Torbjørn Elvsåshagen, Shahram Bahrami, Zillur Rahman, Alexey Shadrin, Daniel E. Askeland-Gjerde, Dennis van der Meer, Oleksandr Frei, Tobias Kaufmann, Ida E. Sønderby, Sigrun Halvorsen, Lars T. Westlye, Ole A. Andreassen

**Affiliations:** 1 Norwegian Centre for Mental Disorders Research (NORMENT), Division of Mental Health and Addiction, Oslo University Hospital and University of Oslo, 0424 Oslo, Norway; 2 Section for Precision Psychiatry, Division of Mental Health and Addiction, Oslo University Hospital, 0424 Oslo, Norway; 3 Institute of Clinical Medicine, University of Oslo, 0318 Oslo, Norway; 4 Department of Neurology, Oslo University Hospital, Oslo, Norway; 5 Department of Behavioural Medicine, Institute of Basic Medical Sciences, Faculty of Medicine, University of Oslo, 0317 Oslo, Norway; 6 Center for Precision Psychiatry, Division of Mental Health and Addiction, Institute of Clinical Medicine, University of Oslo, 0424 Oslo, Norway; 7 School of Mental Health and Neuroscience, Faculty of Health, Medicine and Life Sciences, Maastricht University, Maastricht 6200 MD, the Netherlands; 8 Department of Psychiatry and Psychotherapy, University of Tübingen, Tübingen, Germany; 9 German Center for Mental Health (DZPG), Partner Site Tübingen, Tübingen, Germany; 10 Department of Medical Genetics, Oslo University Hospital, 0424 Oslo, Norway; 11 Department of Cardiology, Oslo University Hospital Ullevål and University of Oslo, 0424 Oslo, Norway; 12 Department of Psychology, University of Oslo, 0373 Oslo, Norway

**Keywords:** brain structure, magnetic resonance imaging, MRI, morphology, hypertensive, genotype, linear regression, advanced statistical tool, genetic overlap, subregion

## Abstract

While brainstem regions are central regulators of blood pressure, the neuronal mechanisms underlying their role in hypertension remain poorly understood. Here, we investigated the structural and genetic relationships between global and regional brainstem volumes and blood pressure. We used magnetic resonance imaging data from *n* = 32,666 UK Biobank participants, and assessed the association of volumes of the whole brainstem and its main regions with blood pressure. We applied powerful statistical genetic tools, including bivariate causal mixture modeling (MiXeR) and conjunctional false discovery rate (conjFDR), to non-overlapping genome-wide association studies of brainstem volumes (*n* = 27,034) and blood pressure (*n* = 321,843) in the UK Biobank cohort. We observed negative associations between the whole brainstem and medulla oblongata volumes and systolic blood and pulse pressure, and positive relationships between midbrain and pons volumes and blood pressure traits when adjusting for the whole brainstem volume (all partial correlation coefficients ∣*r*∣ effects between 0.03 and 0.05, *p* ≤ 0.0042). We observed the largest genetic overlap for the whole brainstem, sharing 77% of its trait-influencing variants with blood pressure. We identified 65 shared loci between brainstem volumes and blood pressure traits and mapped these to 71 genes, implicating molecular pathways linked to sympathetic nervous system development, metal ion transport, and vascular homeostasis. The present findings support a link between brainstem structures and blood pressure and provide insights into their shared genetic underpinnings. The overlapping genetic architectures and mapped genes offer mechanistic information about the roles of brainstem regions in hypertension.

## Teaser

We report structural and genetic links between the brainstem and blood pressure traits. We identified 65 shared loci between brainstem volumes and blood pressure traits and mapped these to 71 genes. The findings provide insights into shared genetic pathways and mechanisms linking brainstem and blood pressure traits.

## Introduction

Hypertension is one of the leading contributors to ischemic heart disease, heart failure, and stroke and accounts for at least 13% of deaths worldwide.^1^^,^^2^^,^^3^^,^^4^ The pathophysiology of chronic blood pressure elevation remains poorly understood, and only half of those with hypertension have adequate blood pressure control.^5^^,^^6^

The brainstem is a regulator of blood pressure^7^^,^^8^ and includes the midbrain, pons, and medulla oblongata. Disentangling the role of the brainstem in the neuronal networks underlying human blood pressure regulation is one critical step toward more effective treatments and improved outcomes in hypertension. The evidence for neuronal involvement in blood pressure regulation comes primarily from animal models.^9^ Invasive animal studies suggest that nuclei within brainstem regions have central roles in regulating sympathetic nerve activity, which is a major contributor to the hypertensive state.^9^^,^^10^^,^^11^ Of particular interest for understanding sympathetic nerve tone are neurons within the rostral ventrolateral medulla oblongata and midbrain; these are critical for experimental hypertension in rodents.^8^^,^^12^ We know less about the roles of brainstem regions in human blood pressure regulation. Some recent fMRI studies assessed brainstem nuclei activity in humans in response to pharmacological stimulation, orthostatic stress, and hypoxia and suggested that several nuclei are part of the central cardiovascular network.^13^^,^^14^^,^^15^ However, there is no previous investigation of whether and how the structure of these regions influences blood pressure in humans.

The unprecedented availability of a large neuroimaging dataset from the UK Biobank^16^ and the development of a brainstem segmentation algorithm^17^ allowed us to identify genetic loci linked to the structure of individual brainstem regions through genome-wide association studies (GWASs).^18^ Prior GWASs have also shed light on the genetic underpinnings of human blood pressure^19^^,^^20^^,^^21^ and recently identified 505 independent loci linked to blood pressure traits.^21^ Despite the substantial genetic contributions to brainstem structure and blood pressure variation and their putative mechanistic links, the genetic relations between brainstem regions and blood pressure traits are largely unknown.

The present study investigated the phenotypic and genetic relationships between global and sub-regional brainstem volumes and blood pressure. To this end, we tested the associations between MRI-based brainstem volumes and blood pressures of UK Biobank participants (*n* = 32,666). We performed follow-up analyses for age- and sex-related patterns. Next, to characterize the overlap in genetic architectures, we applied the bivariate causal mixture model (MiXeR)^22^^,^^23^ and the conjunctional false discovery rate (conjFDR) method^24^^,^^25^ to non-overlapping GWASs of brainstem volumes (*n* = 27,034)^18^ and blood pressure metrics (*n* = 321,843) in the UK Biobank cohort. To further validate the genetic overlap results, we also applied MiXeR and conjFDR to the brainstem data and GWAS summary statistics of blood pressure measures from 318,891 participants from the Million Veteran Program (MVP) consortium.^21^ MiXeR estimates the total number of trait-influencing variants for a given trait and the total number of shared and unique trait-influencing variants for a pair of traits.^22^^,^^23^ ConjFDR enables the detection of individual genetic loci jointly associated with two phenotypes.^24^^,^^25^

## Material and methods

### Study design and participants

For the structural analyses, we included 32,666 UK Biobank participants (53.8% women) with brain MRI and blood pressure data. We excluded participants who did not pass quality control (see below) or withdrew their informed consent (opt-out list dated August 9, 2021). The UK Biobank obtained informed consent from all participants, has institutional review board approval from the North West Multi-center Research Ethics Committee, and the Ethics Advisory Committee (https://www.ukbiobank.ac.uk/ethics) oversees the UK Biobank Ethics and Governance Framework.^16^ We obtained access to the UK Biobank cohort through application no. 27412. We have approval from the Regional Committees for Medical and Health Research Ethics in Norway.

For the primary genetic overlap analyses, we ran GWASs of blood pressure traits for 321,843 white European participants from the UK Biobank cohort. We included our previous GWAS summary statistics of brainstem volumes of 27,034 UK Biobank participants.^18^ For the blood pressure GWASs, we excluded UK Biobank participants who were part of the brainstem GWASs.

Moreover, to further validate the genetic overlap results, we also used GWAS summary statistics of the blood pressure measures from 318,961 participants from the MVP consortium accessed through dbGAP under accession no. phs001672.v9.p1. The MVP consortium obtained consent from all participants and followed all relevant ethical regulations.^21^

### Demographic and clinical data

We extracted demographic data (age, sex, self-reported ethnicity) and systolic and diastolic blood pressure from the UK Biobank repository. We extracted potential confounders and relevant phenotypic variables, based on our previous research,^26^ including body anthropometrics (waist circumference, hip circumference, body mass index), self-identified ethnicity, and variables reflecting cardiovascular risk (i.e., self-reported history of diabetes, hypercholesterolemia, hypertension, current cigarette smoker, and current alcohol consumption).

For blood pressure, we computed the average of two subsequent automatic blood pressure readings when available for systolic and diastolic blood pressure. If only a single automatic blood pressure reading was available, then we used the single measurement. If no automatic readings were available, then we would similarly use the manual blood pressure assessment. Subsequently, we computed pulse pressure as the difference between systolic and diastolic blood pressure. Additionally, we computed waist-to-hip ratio (WHR) as waist circumference divided by hip circumference. Based on the available data, we derived a binary variable for self-identified white European (white, white British, white Irish, other white background)/non-European (otherwise) ethnicity. See Note S1 for the extracted UK Biobank data-field identifiers (IDs) and additional data processing details.

### MRI acquisition, processing, and quality control

The participants’ inclusion was performed at three different sites (Cheadle, Reading, or Newcastle), and all participants underwent 3T brain MRI using similar scanners and protocols.^16^ All data for each participant, including MRI and blood pressure traits, were collected on the same day and site.

We have previously described the processing of brain MRI in detail.^18^ Briefly, we processed brain MRI DICOM (Digital Imaging and Communications in Medicine) data in-house using FreeSurfer^27^ (version 5.3.0; http://www.freesurfer.net), followed by Bayesian brainstem segmentation and delineation of the whole brainstem, midbrain, pons, superior cerebellar peduncle, and medulla oblongata using FreeSurfer (version 6.0).^17^ We extracted the whole brainstem, midbrain, pons, and medulla oblongata and estimated intracranial volume (ICV),^28^ and Euler numbers^29^ as a proxy for image quality^30^ for the statistical analyses.

We applied MRI quality assessment using Euler numbers.^30^ Among the 42,063 participants with available brain MRI, we removed 3 with missing Euler numbers and 4,206 based on a previously outlined procedure.^26^ Then, we removed the remaining participants who did not pass manual MRI quality control,^18^ further excluding 48 participants. Lastly, we removed 5,140 participants with incomplete demographic and clinical data needed for the statistical analyses. This resulted in the final sample of 32,666 participants.

### Statistical analysis of brainstem volumes

We investigated the sample demographics and brainstem volumes across and within sexes. We compared categorical variables using the χ^2^ test. For normally or non-normally distributed continuous variables, we used the two-sample t test or the Wilcoxon rank-sum test, respectively. For unequal variance across sexes, we replaced the t test with the Welch approximation. We verified normality by visually evaluating density plots (Figures S1 and S2).

We used multiple linear regression to investigate the association between blood pressure traits and the whole brainstem and its main regions. We adjusted for age, age^2^, sex, age-by-sex, age^2^-by-sex, WHR, WHR^2^, ethnicity, and metabolic/lifestyle variables (including current cigarette smoking [yes/no], current alcohol consumption [yes/no], diabetes [yes/no], and hypercholesterolemia [yes/no]), ICV, image quality (average Euler number), and site. We adjusted for age^2^ to account for age-related nonlinearities and WHR and WHR^2^ since we have previously shown that WHR is nonlinearly associated with the whole brainstem.^26^ The analyses for brainstem regions were additionally adjusted for whole brainstem volume, thus revealing associations with blood pressure beyond global brainstem volume, analogous to recent studies.^18^^,^^31^^,^^32^ We entered categorical variables as factors and mean-centered all continuous variables.

We performed follow-up analyses in the participants with a normal blood pressure range, excluding those with hypertension (defined here as self-reported hypertension or systolic blood pressure ≥140 mm Hg or diastolic blood pressure ≥90 mm Hg on the day of study participation). Lastly, we investigated age- and sex-related patterns by conducting separate analyses for participants aged <60 years and for participants aged ≥60 years and for men and women. The supplement includes complementary analyses of brainstem regions without adjustment for whole brainstem volume (Note S2).

We performed statistical analyses in R (version 3.6.0; https://www.r-project.org). We computed the partial correlation coefficients, *r*, effect size directly from the t statistics for continuous variables and via Cohen’s d for categorical variables.^33^ We evaluated model residuals for normality using residual vs. fitted value and quantile-quantile (Q-Q) plots and decided to move forward without log transformation of any regression model variables. We used Bonferroni correction to adjust for multiple comparisons at α = 0.05 across N_1_ = 12 independent tests (i.e., the number of linear regression models, counting partly overlapping models once, reflecting the included four brain structures and three blood pressure traits, yielding a significance threshold of *p* ≤ α/N_1_ = 0.0042, rounded to four decimal points, for the phenotypic brainstem volume analyses).

### GWASs, genetic overlap analyses, and functional annotation

We first conducted GWASs for systolic and diastolic blood pressure and pulse pressure in 321,843 unrelated (king-cutoff 0.05) white British participants (53.9% women) from the UK Biobank, after excluding those who were part of the brainstem GWASs. We used PLINK2 to perform general linear models in 1,1172,639 SNPs filtered with minor allele frequency = 0.005 and minor allele count = 20. Then, for the primary genetic overlap analyses, we applied MiXeR and conjFDR to these UK Biobank GWAS data and the GWASs summary statistics of brainstem volumes (*n* = 27,034).^18^ These analyses were also run separately for women and men.

Next, we applied the linkage disequilibrium (LD) score regression (LD Score version 1.0.1) method^34^^,^^35^ to determine the genetic correlation (r_g_) between summary statistics of blood pressure traits and brainstem volumes. Subsequently, we applied the bivariate causal mixer model (MiXeR)^22^^,^^23^ to the same GWAS summary statistics. MiXeR is a Bayesian statistical genetics tool that characterizes the pattern of genetic overlap between two complex phenotypes. The tool uses summary statistics data from two GWASs to estimate the number of trait-influencing genetic variants (polygenicity) and the variance of their effect sizes (discoverability) for each of the two analyzed traits and to quantify proportion of variants shared between two phenotypes. Association signals observed in the GWAS are refined by modeling a fine-grained correlation structure between genetic variants induced by LD and accounting for sample overlap between analyzed GWASs. Variant effect sizes are modeled with point-normal mixture priors, where variants shared between phenotypes are allowed to have both concordant and discordant directions of effects. We present the results of the MiXeR analysis as Venn diagrams, illustrating the proportion of shared and trait-specific trait-influencing SNPs. We evaluated the model fit based on predicted vs. observed conditional Q-Q plots (a closer fit between the dashed and continuous line of the same color suggests a better fit) and log likelihood plots that visualize the parameter estimation procedure (Figures S3–S5). Additionally, we used the Akaike information criterion (AIC) to compare each model fitted using MiXeR with two competitive models: (1) the model with maximum possible genetic overlap between two analyzed phenotypes (when all variants influencing the phenotype with smaller polygenicity also influence the phenotype with larger polygenicity, resulting in embedded circles in a Venn diagram) and (2) the model with minimum possible genetic overlap between two analyzed phenotypes for a given level of genetic correlation between them, as defined by Frei et al.^22^ The positive difference between the AIC value of the competitive model and the AIC value of the MiXeR model (AIC_competitive − AIC_MiXeR) indicates that the MiXeR model provides a better fit to the observed GWAS data and should be prioritized over the competitive model. To provide convergent evidence of genetic overlap between analyzed phenotypes from different methods, we also deployed for this purpose conditional Q-Q plots.^24^ Conditional Q-Q plots is an extension of the standard Q-Q plots commonly applied in GWASs. Standard Q-Q plots depict the quantiles of the association *p* values observed in the GWAS on the y axis against the theoretical quantiles expected under the global null hypothesis (none of the SNPs is associated with the phenotype) on the x axis. Both observed and theoretical *p* value quantiles are commonly plotted on the −log10 scale. In the case of no association, a Q-Q plot follows a straight (null) line but deflects leftward from this null line when some form of systematic association is present. Conditional Q-Q plots are constructed by creating subsets of SNPs based on the level of association with the secondary (conditional) phenotype. A standard Q-Q plot of each SNP subset is then plotted. Cross-trait enrichment can be seen as successive leftward deflections of the Q-Q plots for SNP subsets with increasing levels of significance in the secondary phenotype.

Following MiXeR, we used conjFDR^24^^,^^25^ to determine shared genetic loci between two traits. Briefly, conjFDR is an extension of the conditional FDR (condFDR). The condFDR method takes GWAS summary statistics on two phenotypes: a phenotype of interest (primary) and a secondary (conditional) phenotype. For a given variant condFDR uses its *p* values for association with primary and conditional phenotypes to estimate a posterior probability that the variant is null (has no association) in the primary phenotype. Employing pleiotropy with the conditional phenotype results in re-ranking of variants compared to the ranking based on *p* values from the GWAS on the primary phenotype. In contrast, ranking variants based on unconditional FDR (e.g., using the Benjamini-Hochberg procedure) does not change their order (compared to the ordering based on GWAS *p* values). It was shown that by leveraging associations with a secondary phenotype, condFDR method increases the power to discover loci associated with a primary phenotype.^36^ The conjFDR is defined as the maximum of the two condFDR values obtained by inverting the roles of the primary and secondary phenotypes, providing a conservative estimate of the FDR for an SNP association with both phenotypes jointly. We considered loci significant if the lead SNP passed the genome-wide significance threshold at conjFDR <0.05. To avoid potential biases due to complex LD structures, we performed the analysis after excluding SNPs in the extended major histocompatibility complex (MHC; hg19 location chr6: 25119106–33854733) and 8p23.1 (hg19 location chr8: 7242715–12483982) regions.

We used the Open Targets platform to map the significant SNPs to genes. Open Targets maps SNPs to genes by combining positional information regarding the distance between the variant and each gene’s canonical transcription start site, expression quantitative trait locus (QTL), protein QTL, splicing QTL, epigenomic data, and functional prediction.^37^ After excluding genes in the MHC region, we applied gene-set analyses with a Bonferroni correction on all mapped genes using FUMA.^38^

To further validate the genetic overlap results, we applied the MiXeR and conjFDR to the summary statistics of the UK Biobank brainstem volumes and blood pressure traits of the MVP cohort (*n* = 318,891).^21^ Finally, we conducted binomial tests to assess the sign concordance between the primary genetic overlap analyses (i.e., UK Biobank brainstem volumes vs. UK Biobank blood pressure metrics) and the genetic overlap analyses that used the MVP cohort (i.e., UK Biobank brainstem volumes vs. MVP blood pressure metrics).

## Results

### Demographic and clinical data

Table S1 summarizes the demographic and clinical data. The final sample (*n* = 32,666) included more women (*n* = 17,561; 53.8%) than men (*n* = 15,105; 46.2%) and comprised mainly self-identified white European ethnicity (97.0%). The age range was 44–82 years; on average, the women were slightly younger than men (mean ± SD ages 62.5 ± 7.3 and 63.6 ± 7.6 years, respectively). Men had, as expected,^26^ generally higher levels of adverse cardiometabolic factors than women. Additionally, men had larger volumes of the whole brainstem and brainstem regions and higher systolic and diastolic blood pressure and pulse pressure than women (Table S2; Figure S2).

### Brainstem volumes and blood pressure traits

**Figure 1:**
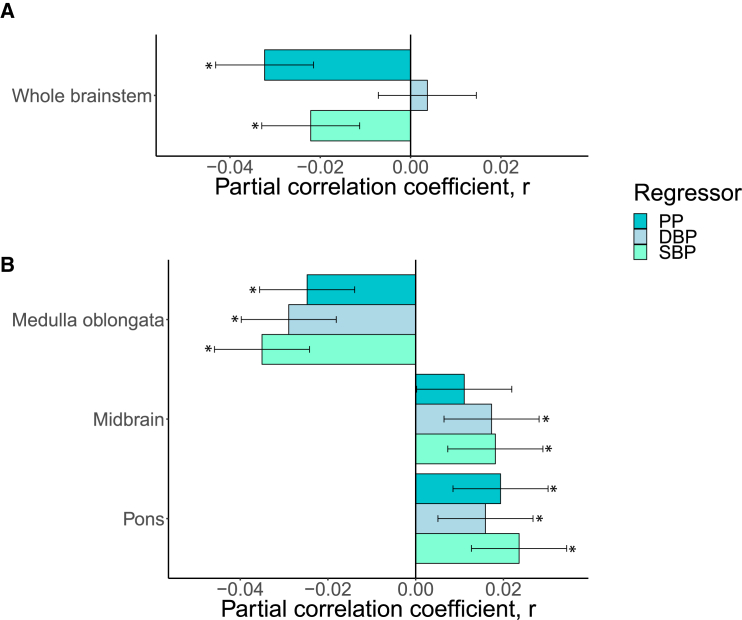
Blood pressure and brainstem volumes We used multiple linear regression to investigate the association between blood pressure metrics and volumes of the (A) whole brainstem and (B) brainstem regions (*n* = 32,666). We adjusted for age, age^2^, sex, age-by-sex, age^2^-by-sex, WHR, WHR^2^, ethnicity, and metabolic/lifestyle variables (including current cigarette smoking [yes/no], current alcohol consumption [yes/no], diabetes [yes/no], and hypercholesterolemia [yes/no]), ICV, image quality (average Euler number), and site. Brainstem regions were additionally adjusted for the whole brainstem. The error bars indicate the 95% confidence interval of the r effect size. DBP, diastolic blood pressure; PP, pulse pressure; SBP, systolic blood pressure.

The multiple linear regression analysis revealed significant negative associations between the whole brainstem volume and pulse pressure (*r* = −0.03, *p* = 5.1 × 10^−9^) and systolic blood pressure (*r* = −0.02, *p* = 6.4 × 10^−5^) (Figure 1A; Table S3A). Medulla oblongata volume was negatively associated with pulse pressure (*r* = −0.02, *p* = 7.8 × 10^−6^) and diastolic (*r* = −0.03, *p* = 1.7 × 10^−7^) and systolic (*r* = −0.04, *p* = 2.4 × 10^−10^) blood pressure. Pons volume was positively associated with pulse pressure (*r* = 0.02, *p* = 0.0004) and diastolic (*r* = 0.02, *p* = 0.0040) and systolic (*r* = 0.02, *p* = 2.1 × 10^−5^) blood pressure. Midbrain volume was positively associated with systolic (*r* = 0.02, *p* = 0.0010) and diastolic (*r* = 0.02, *p* = 0.0018) blood pressure (Figure 1B; Table S3B). We observed no other significant associations between brainstem volumes and blood pressure traits.

Moreover, when the analyses for brainstem regions were run without adjusting for the whole brainstem, there were significant negative associations between medulla oblongata volumes and systolic blood pressure (*r* = −0.04, *p* = 1.4 × 10^−12^) and pulse pressure (*r* = −0.04, *p* = 1.4 × 10^−13^), pons volume was negatively associated with pulse pressure (*r* = −0.03, *p* = 1.0 × 10^−6^) and systolic blood pressure (*r* = −0.02, *p* = 0.0035), and midbrain volume was negatively associated with pulse pressure (*r* = −0.02, *p* = 2.6 × 10^−5^) (Table S3A; Note S2).

In follow-up analyses of non-hypertensive participants (*n* = 15,870; 61.4% women), we found a similar association pattern as in the full sample, albeit with fewer significant findings, likely due to the smaller sample (Figure S6; Tables S4A and S4B).

### Age-related patterns

For those aged 60 and above (*n* = 21,697; 52.2% women), we observed similar significant relationships between brainstem volumes and blood pressure traits as in the primary regression analyses (Figures S7A and S7B; Tables S5A and S5B). In participants younger than 60 years (*n* = 10,969; 57.0% women), we observed a similar pattern of relationships but no significant findings (Figures S7C and S7D; Tables S6A and S6B).

### Sex-related patterns

**Figure 2:**
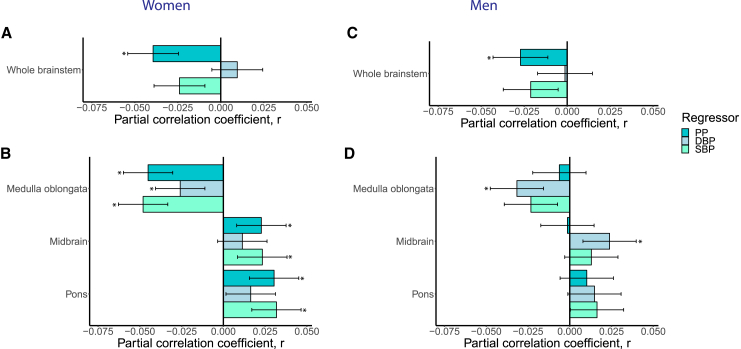
Sex-specific patterns of blood pressure and the brainstem We used multiple linear regression to separately investigate the association between blood pressure metrics and volumes of (A) the whole brainstem and (B) brainstem regions in women (*n* = 17,561) and (C) the whole brainstem and (D) brainstem regions in men (*n* = 15,105). We adjusted for age, age^2^, sex, age-by-sex, age^2^-by-sex, WHR, WHR^2^, ethnicity, and metabolic/lifestyle variables (including current cigarette smoking [yes/no], current alcohol consumption [yes/no], diabetes [yes/no], and hypercholesterolemia [yes/no]), ICV, image quality (average Euler number), and site. The error bars indicate the 95% confidence interval of the r effect size. Brainstem regions were additionally adjusted for the whole brainstem.

In separate regression analyses, we observed similar relationships between the whole brainstem volume and blood pressure traits in women (*n* = 17,561) and men (*n* = 15,105), with significantly lower volume at higher pulse pressure (*r* = −0.04 and *p* = 1.8e−7 for women and *r* = −0.03 and *p* = 0.0008 for men) (Figure 2; Tables S7A, S7B, S8A, and S8B). For brainstem regions, women showed significantly lower medulla oblongata volume at higher pulse pressure (*r* = −0.05, *p* = 2.3e−9), diastolic (*r* = −0.03, *p* = 0.0006) and systolic (*r* = −0.05, *p* = 1.9e−10) blood pressure, higher midbrain volume at higher systolic blood pressure (*r* = 0.02, *p* = 0.0020) and pulse pressure (*r* = 0.02, *p* = 0.0026, and higher pons volume at higher pulse pressure (*r* = 0.03, *p* = 5.8e−5) and systolic blood pressure (*r* = 0.03, *p* = 2.6e−5). For men, our findings suggest significantly lower medulla oblongata volume at higher diastolic blood pressure (*r* = −0.03, *p* = 0.0001) and higher midbrain volume at higher diastolic blood pressure (*r* = 0.02, *p* = 0.0034).

### Genetic overlap analyses and functional annotation

First, we found a significant positive genetic correlation between the whole brainstem and diastolic blood pressure (r_g_ = 0.06, SE = 0.03, *p* = 0.046; Table S9) using LD Score regression but observed no other significant genetic correlations.

**Figure 3:**
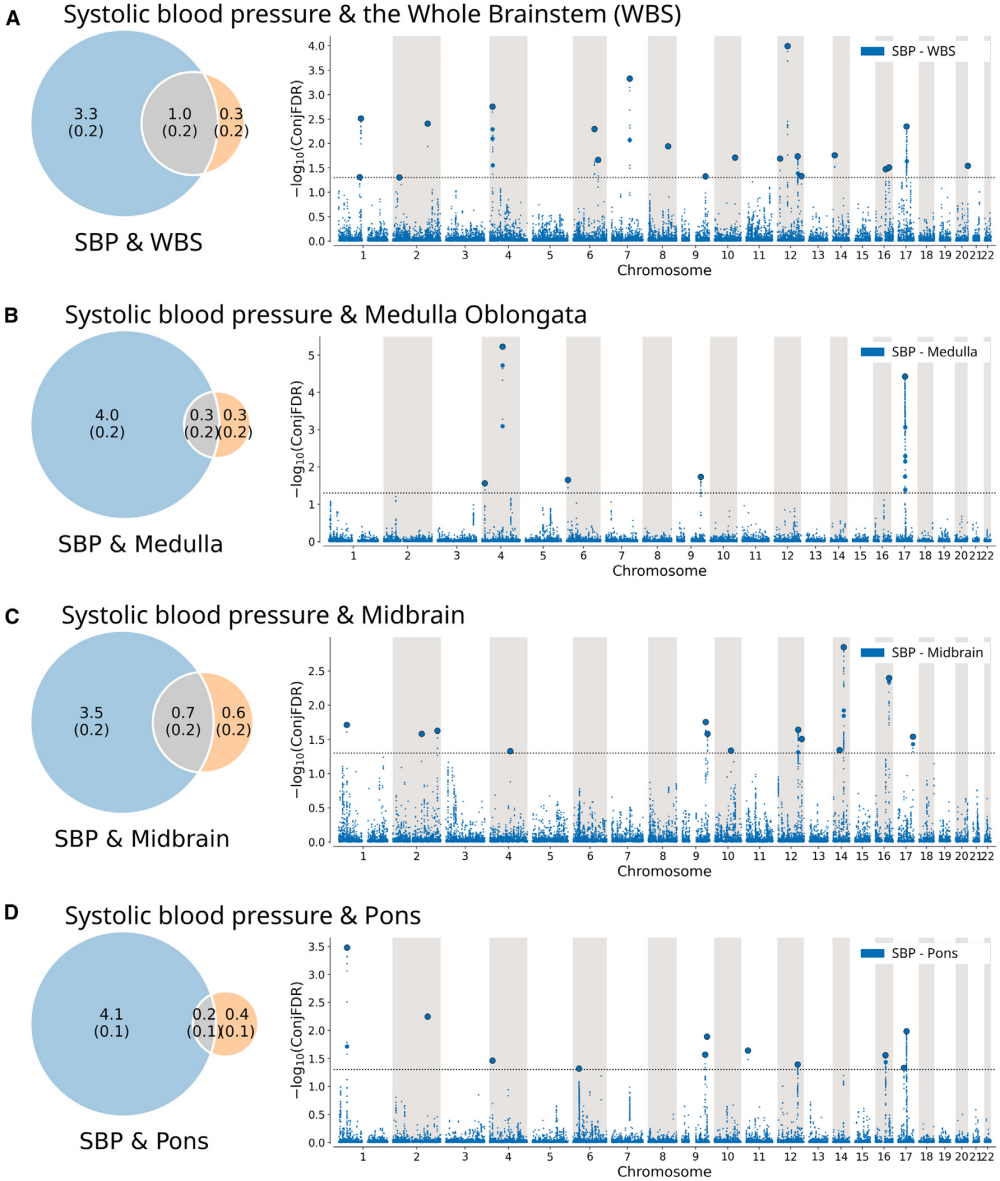
Genetic overlap between systolic blood pressure and brainstem volumes The figure shows the (left) Venn diagrams of the polygenicity of blood pressure metrics (blue) and brainstem regions (orange) and the polygenic overlap between traits (gray), and (right) Manhattan plots of shared loci between pulse pressure and (A) the whole brainstem, (B) medulla oblongata, (C) midbrain, and (D) pons. Venn diagrams illustrate the estimated number of trait-influencing variants shared (gray) between the blood pressure metrics (left circle) and brainstem regions (right circle) and unique to either of them (blue for blood pressure metrics and orange for brainstem regions). The number of trait-influencing variants in thousands is shown, with the SE provided in thousands in parentheses. The size of the circles reflects the polygenicity of each phenotype, with larger circles corresponding to greater polygenicity and vice versa. In Manhattan plots, the x axis is chromosomal position, and the y axis shows the significance (–log10(conjFDR)) of association derived by conjFDR. The horizontal dotted black line shows the genome-wide significance level (conjFDR = 0.05). Bold dots with the black outline show lead SNPs of the identified loci; bold dots without the outline show LD-independent (*r*^*2*^ < 0.6) significant associations in the locus. Medulla, edulla oblongata; WBS, whole brainstem.

**Figure 4:**
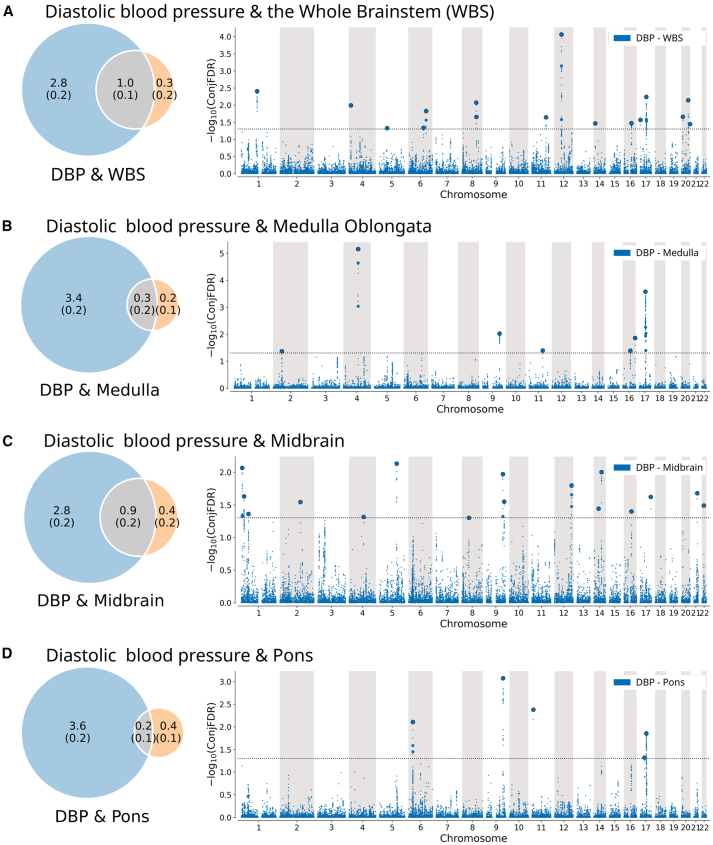
Genetic overlap between diastolic blood pressure and brainstem volumes The figure shows the (left) Venn diagrams of the polygenicity of blood pressure metrics (blue) and brainstem regions (orange) and the polygenic overlap between traits (gray) and (right) Manhattan plots of shared loci between pulse pressure and (A) the whole brainstem, (B) medulla oblongata, (C) midbrain, and (D) pons. Venn diagrams illustrate the estimated number of trait-influencing variants shared (gray) between the blood pressure metrics (left circle) and brainstem regions (right circle) and unique to either of them (blue for blood pressure metrics and orange for brainstem regions). The number of trait-influencing variants in thousands is shown, with the SE provided in thousands in parentheses. The size of the circles reflects the polygenicity of each phenotype, with larger circles corresponding to greater polygenicity and vice versa. In Manhattan plots, the x axis is the chromosomal position, and the y axis shows the significance (–log10(conjFDR)) of association derived by conjFDR. The horizontal dotted black line shows the genome-wide significance level (conjFDR = 0.05). Bold dots with the black outline show lead SNPs of the identified loci; bold dots without the outline show LD-independent (*r*^*2*^ < 0.6) significant associations in the locus.

**Figure 5:**
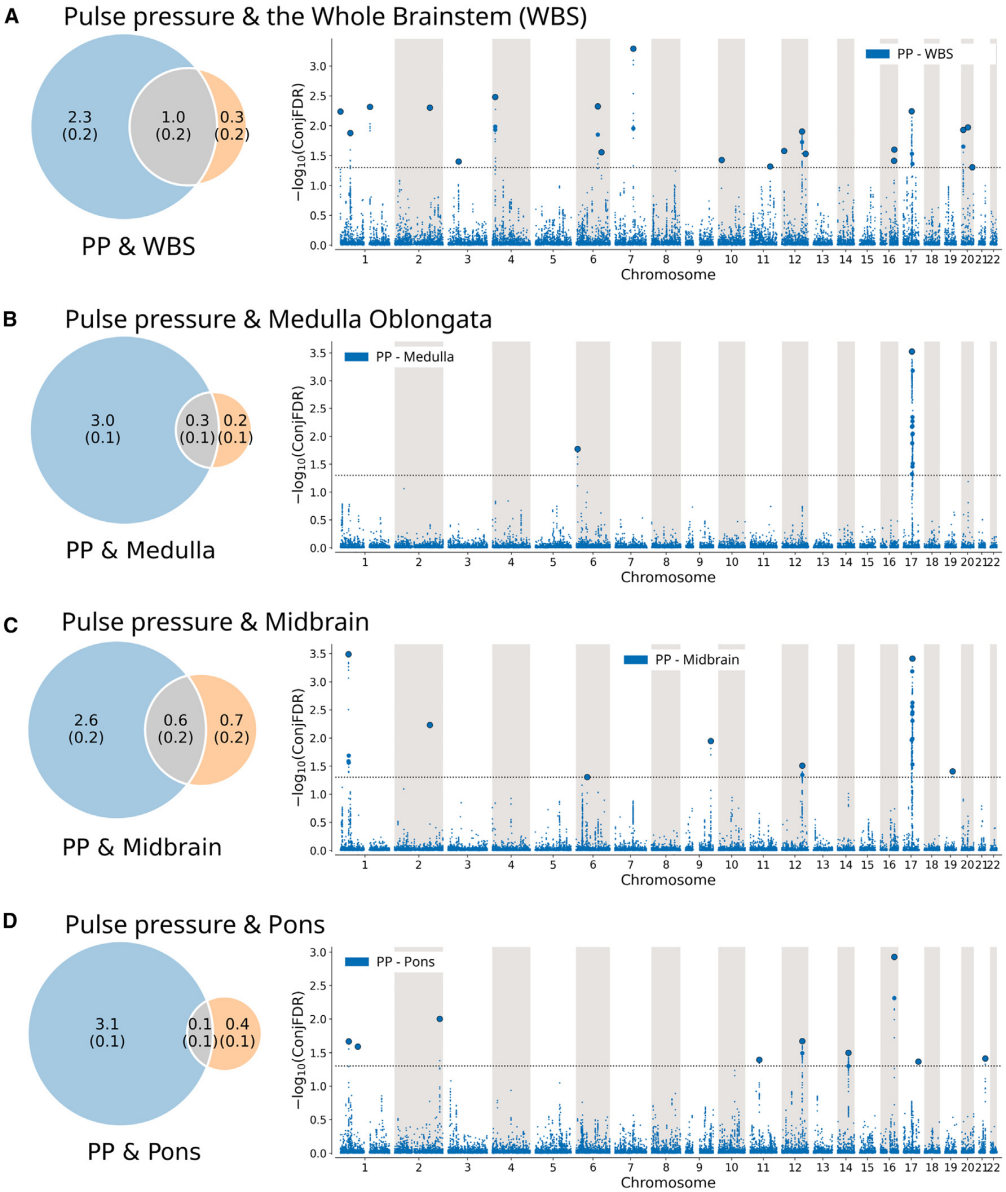
Genetic overlap between pulse pressure and brainstem volumes The figure shows the (left) Venn diagrams of the polygenicity of blood pressure metrics (blue) and brainstem regions (orange), and the polygenic overlap between traits (gray) and (right) Manhattan plots of shared loci between pulse pressure and (A) the whole brainstem, (B) medulla oblongata, (C) midbrain, and (D) pons. Venn diagrams illustrate the estimated number of trait-influencing variants shared (gray) between the blood pressure metrics (left circle) and brainstem regions (right circle) and unique to either of them (blue for blood pressure metrics and orange for brainstem regions). The number of trait-influencing variants in thousands is shown, with the SE provided in thousands in parentheses. The size of the circles reflects the polygenicity of each phenotype, with larger circles corresponding to greater polygenicity and vice versa. In Manhattan plots, the x axis is the chromosomal position, and the y axis shows the significance (–log10(conjFDR)) of association derived by conjFDR. The horizontal dotted black line shows the genome-wide significance level (conjFDR = 0.05). Bold dots with the black outline show lead SNPs of the identified loci; bold dots without the outline show LD-independent (*r*^*2*^ < 0.6) significant associations in the locus.

Second, using univariate MiXeR, we observed that blood pressure is more polygenic than the brainstem. As illustrated by the Venn diagrams in Figures 3, 4, and 5, the number of trait-influencing variants for systolic blood pressure, diastolic blood pressure, and pulse pressure were up to 4–6 times as high (4.3, 3.8, and 3.3 K for systolic, diastolic, and pulse pressure, respectively) as for brainstem regions (1.3, 1.3, 0.6, and 0.6 K for the whole brainstem, midbrain, pons, and medulla oblongata, respectively).

The bivariate MiXeR analysis revealed polygenic overlap between brainstem regions and blood pressure. The highest estimated number of shared trait-influencing variants are between the whole brainstem and systolic blood pressure (1.0 K; i.e., 77% of all whole brainstem variants), diastolic blood pressure (1.0 K; i.e., 77% of all whole brainstem variants), and pulse pressure (1.0 K; i.e., 77% of all whole brainstem variants), as illustrated by the intersection of corresponding Venn diagrams (Figures 3, 4, and 5). We observed the second highest estimated number of shared trait-influencing variants between the midbrain and systolic blood pressure (0.7 K; i.e., 54% of the midbrain variants), diastolic blood pressure (0.9 K; i.e., 69% of the midbrain variants), and pulse pressure (0.6 K; i.e., 40% of the midbrain variants). The estimated numbers for trait-influencing variants shared between the pons and medulla oblongata and blood pressure traits were lower and ranged between 0.1 and 0.3 K. MiXeR was also run for women and men separately and showed similar, consistent patterns of overlap for the two sexes (Figures S8–S10).

Third, we observed successive increments of SNP enrichment for the whole brainstem and its regions when conditioned on association *p* values for blood pressure traits on conditional Q-Q plots of the condFDR analysis (Figures S11–S13). The consistently increasing leftward deflection for subsets of variants with higher significance in the conditional trait in both directions indicates substantial polygenic overlap between the brainstem and blood pressure traits.

Fourth, we revealed 65 loci shared between the brainstem volumes and blood pressure traits using the conjFDR analysis (Tables S10–S27) and illustrated the observed genetic overlaps using Manhattan plots (Figures 3, 4, and 5). We identified 34, 36, and 31 shared loci between systolic blood, diastolic blood, and pulse pressure, and the whole brainstem and its regions, respectively. Specifically, we identified (1) 20, 12, 11, and 5 loci shared between systolic blood pressure and the whole brainstem, midbrain, pons, and medulla oblongata, respectively (Figure 3); (2) 16, 15, 5, and 7 loci were jointly associated with diastolic blood pressure and the whole brainstem, midbrain, pons, and medulla oblongata, respectively (Figure 4); and (3) 20, 9, 7, and 2 loci were shared between pulse pressure and the whole brainstem, midbrain, pons, and medulla oblongata, respectively (Figure 5). For both systolic and diastolic blood pressure, we observed the overall most significant genetic overlaps for medulla oblongata on chromosome 4, followed by the medulla oblongata and chromosome 5. For pulse pressure, we observed the overall most significant results for medulla oblongata and chromosome 17 and the overall largest number of shared loci for the whole brainstem, with the most significant findings for chromosomes 1, 12, and 20.

The conjFDR analysis was then run for women and men separately. The number of shared loci for women, men were for systolic blood pressure and the whole brainstem (10, 8), midbrain (3, 5), pons (4, 2), and medulla oblongata (4, 2); for diastolic blood pressure and the whole brainstem (7, 4), midbrain (5, 5), pons (4, 1), and medulla oblongata (6, 1); and for pulse pressure and the whole brainstem (7, 4), midbrain (5, 5), pons (4, 1), and medulla oblongata (6, 1; Tables S28–S51).

Next, we mapped all lead SNPs for each shared locus from conjFDR analyses to genes using the Open Targets platform (Table S23). We observed 36, 37, and 33 mapped genes between systolic blood, diastolic blood, and pulse pressure with brainstem regions. Subsequently, we performed gene-set analyses on all the mapped genes. We observed significant enrichment for five gene set terms when mapped with genes for the whole brainstem and diastolic blood pressure, one gene set for the whole brainstem and pulse pressure, one gene set for midbrain and systolic blood pressure, and one gene set for medulla oblongata and pulse pressure (Tables S24–S27). We observed the most significant gene set for whole brainstem and diastolic blood pressure (*p* = 8.5e−7); this gene set is linked to brain morphology. The latter gene set includes the JAGGED1 (*JAG1* [MIM: 609920]) and the FORKHEAD BOX O3A (*FOXO3A* [MIM: 602681]) genes and these were also part of several of the other enriched gene sets. *JAG1* and *FOXO3A* have been associated with neurodevelopmental abnormalities and vascular homeostasis, respectively.^39^^,^^40^ The BONE MORPHOGENETIC PROTEIN 4 (*BMP4* [MIM: 112262]) and the BONE MORPHOGENETIC PROTEIN 6 (*BMP6* [MIM: 112266]) genes were also represented in several of the significantly enriched gene sets. Notably, the *BMP4* and *BMP6* genes have been linked to sympathetic nervous system development.^41^

Lastly, we applied MiXeR and conjFDR to the UK Biobank brainstem volumes and the MVP blood pressure metrics (Tables S52–S63). Here, MiXeR showed overlap in line with the primary genetic analyses, and the conjFDR revealed 42 unique loci shared between the brainstem volumes and the blood pressure traits. The binomial tests for sign concordance with the primary genetic overlap results (i.e., UK Biobank brainstem volumes vs. UK Biobank blood pressure metrics) were then run (for trait pairs with more than four shared loci), and we found that all but one of these tests were significant (Table S64). Furthermore, across all loci jointly associated with brainstem volumes and blood pressure metrics in the primary genetic analysis, 91% of them had the same effect direction when using data from the MVP cohort.

## Discussion

While the brainstem plays a known role in hypertension, how its structure and genetic architecture are linked to human blood pressure remains largely unknown. Here, we identified negative associations between the whole brainstem and medulla oblongata volumes and blood pressure traits and positive relationships between midbrain and pons volumes and blood pressure when adjusting for whole brainstem volume, leveraging the UK Biobank participants and powerful analytical tools. We also discovered extensive genetic overlap for whole brainstem volume and blood pressure traits, which shared 77% of the trait-influencing variants of the brainstem. Finally, we identified 65 individual genetic loci jointly associated with brainstem structures and blood pressure traits and mapped these to 71 genes, implicating sympathetic nervous system development, metal ion transport, and vascular homeostasis.

The current large-scale neuroimaging study links blood pressure to brainstem structure variation. We observed a consistent pattern linking brainstem volume to blood pressure traits both in middle age and late life. Although some previous work reported positive associations between blood pressure traits and late-life brain volumes,^42^^,^^43^ most studies observed negative associations between brain volumes and blood pressure across adulthood.^44^^,^^45^^,^^46^^,^^47^ These found that higher blood pressure and hypertension were related to lower total brain volume and reduced regional brain volumes, including smaller frontal and medial temporal structures (e.g., the hippocampus).^44^^,^^45^^,^^46^^,^^47^ Furthermore, some studies found that higher blood pressure in early adulthood and midlife was associated with smaller late-life brain volumes.^48^^,^^49^ Our findings suggest a continuous association with small effects between brainstem volumes and blood pressure in non-hypertensive and hypertensive states.

Furthermore, we observed indications of different association patterns between brainstem regions and blood pressure traits in women and men, with predominantly significant associations in women but not men, despite, on average lower blood pressure, general cardiometabolic risk, and age for women relative to men. Indeed, our observations may relate to sex differences in blood pressure regulation, hypertension, and cardiovascular disease, as well as sex hormones^50^^,^^51^^,^^52^ and, for example, body composition, lifestyle, and environmental factors.^52^ To address these observed sex differences fully and how they relate to hypertension disease risk and related markers and outcomes, designated studies are required.

The precise mechanisms underlying the association between blood pressure and brainstem volumes remain to be clarified. On the one hand, higher blood pressure may result in cerebral vessel endothelial dysfunction, arteriolosclerosis, regional hypoperfusion, reduced blood-brain barrier integrity, gray and white matter damage, and possibly brain volume loss.^53^^,^^54^^,^^55^^,^^56^ These alterations could contribute to the negative relationships between blood pressure metrics and volumes of the whole brainstem and medulla oblongata in the present study. However, we also found positive associations between blood pressure and volumes of the midbrain and pons, which is not consistent with blood pressure-induced atrophy. On the other hand, between-subjects variation in brainstem structure (e.g., in tissue volume and number of neurons in blood pressure-linked brainstem nuclei) could influence how the brainstem regulates blood pressure in a given individual.

MiXeR revealed substantial genetic overlap between brainstem volumes and blood pressure. We found the largest number of shared trait-influencing variants for the whole brainstem, which shared 77% of its trait-influencing variants with blood pressure traits. For the brainstem regions, the midbrain showed the greatest overlap, which shared 33%–69% of its trait-influencing variants with blood pressure traits. Despite the substantial genetic overlap, the genetic correlations between brainstem volumes and blood pressure traits were weak (all ∣*r*∣ < 0.07). This pattern of genetic overlap with weak correlation suggests mixed allelic effect directions since the consistent direction of effect across overlapping genetic loci is required for a significant genetic correlation.^35^ In support of this notion, we found that 48% of the 65 individual genetic loci jointly associated with brainstem volumes and blood pressure traits had concordant effects (e.g., greater volume and higher blood pressure). The remaining 52% of the shared loci exhibited discordant effects (e.g., greater volume and lower blood pressure).

The significant associations between the brainstem volumes and blood pressure metrics in the UK Biobank participants and the substantial genetic overlap revealed by MiXeR provide further support for the relevance of brainstem regions in blood pressure regulation. Although the precise neural networks and roles of brain regions in cardiovascular regulation remain to be clarified fully, a large body of evidence implicates several nuclei within the medulla oblongata, midbrain, and pons in blood pressure regulation.^7^^,^^9^^,^^12^ The rostral ventrolateral medulla oblongata is a key regulator of blood pressure and includes the glutamatergic C1 neurons.^7^ These neurons produce adrenaline and project to preganglionic sympathetic and parasympathetic neurons, which in turn innervate the heart, arterioles, and kidneys.^7^^,^^8^^,^^9^ The C1 neurons are thus likely contributors to the excessive sympathetic nerve activity commonly found in hypertension.^7^^,^^9^^,^^12^ Rostral ventrolateral medulla oblongata neurons also project to and receive inputs from other regions within the brainstem linked to blood pressure regulation, such as the periaqueductal gray matter of the midbrain and the locus coeruleus of the pons.^9^ An involvement of midbrain and pontine nuclei in blood pressure regulation is also supported by invasive electrical stimulation studies in individuals with movement disorders; here, electrical stimulation of midbrain^57^ and pontine^58^ nuclei modulated blood pressure.

The current findings provide insight into molecular pathways of hypertension, and several of the shared loci identified by the conjFDR method and the mapped genes are noteworthy. The most significant shared locus for systolic and diastolic blood pressure—rs13107325—was jointly associated with medulla oblongata volume. rs13107325 is in the solute carrier family member 8 (*SLC39A8* [MIM: 608732]) gene and encodes a cellular metal ion transporter. The major allele of *SLC39A8* is highly pleiotropic, associated with increased systolic and diastolic blood pressure,^59^ and is essential for normal development of the heart.^60^ However, the precise mechanisms by which the *SLC39A8* influences blood pressure remain to be clarified. rs17438262 and rs1996656 were jointly associated with systolic blood pressure and pulse pressure and the whole brainstem, midbrain, and pons. These two loci were mapped to the WASH complex subunit 3 (*WASHC3* [MIM: 619925]) gene. *WASHC3* regulates endolysosomal membrane trafficking,^61^ and how the gene may contribute to hypertension mandates further research. We also found that several of the loci jointly associated with brainstem volumes and blood pressure were mapped to *HOX* genes, including HOMEOBOX B3 (*HOXB3* [MIM: 142966]). *HOX* genes encode transcription factors with central roles in nervous system development,^62^ and the *HOXB1–4* genes are critical for the development of the pons and the medulla oblongata.^63^ Interestingly, *HOXB* genes are involved in developing sympathetic neurons, yet how these genes relate to blood pressure regulation remains elusive. *JAG1* and *FOXO3* were represented in the most significant gene set (i.e., for whole brainstem and diastolic blood pressure) and in several of the other enriched gene sets. *JAG1* plays important roles in brain development,^64^ is implicated in the regulation of smooth muscle function in arteries,^65^ and *JAG1* mutations can affect several organs, including the eyes, liver, and heart, and the skeleton.^39^ *FOXO3A* encodes a transcription factor, has been linked to vascular homeostasis and vascular aging, may affect the risk of cerebrovascular disease, and modulates human longevity.^40^ The *BMP4* and the *BMP6* genes were also represented in several of the significantly enriched gene sets. The *BMP* genes are involved in sympathetic nervous system development^41^ and are increasingly recognized for their roles in cardiovascular homeostasis.^66^ Further experimental studies of these overlapping genetic pathways are needed to determine their precise molecular contributions to hypertension.

The MRI segmentation algorithm used in the present study delineates larger regions of the brainstem that each include a substantial number of nuclei—only a subset of which has been linked to blood pressure regulation—and white matter.^17^ Indeed, this is one potential explanation for why the observed associations between brainstem volumes and blood pressure in the UK Biobank participants were small, with significant effect sizes ranging from ∣*r*∣ 0.03 to 0.05. However, new methods for analyzing individual brainstem nuclei were recently developed,^67^^,^^68^ and stronger associations might be revealed in future studies of brainstem structure and blood pressure.

The present study has several strengths and limitations. Concerning the strengths, the current work includes a large sample of genotyped and well-characterized participants with brain MRI and blood pressure data from the UK Biobank. This allowed us to adjust the analyses for several potentially confounding factors, such as body anthropometrics and other cardiovascular risk factors. Furthermore, we used summary statistics from GWASs of blood pressure and brainstem volumes and employed a recently developed and robust method for segmenting brainstem regions. Moreover, we ran separate analyses for women and men and further validated the genetic overlap results using data from the MVP cohort. When it comes to the limitations, there is no other GWAS of midbrain, pons, and medulla oblongata volumes, and this precludes replication analyses. Furthermore, the cross-sectional design and the genetic analyses do not allow for causality assessments. Thus, whether brainstem structure influences blood pressure or vice versa remains to be clarified. However, it is noteworthy that we found significant associations between brainstem volumes and blood pressure, also when excluding those with hypertension. This suggests that hypertension-related atrophy is not the primary mechanism underlying the significant relationships between brainstem structure and human blood pressure. Longitudinal volumetric studies of individual brainstem nuclei and blood pressure traits are needed to elucidate these relationships further.

In conclusion, we observed phenotypic and genetic relationships with small effect sizes between the structure of brainstem regions and blood pressure in humans. The overlapping loci implicated genes involved in neuronal development pathways, metal ion transport, and vascular homeostasis, thus providing insights into the mechanisms of the roles of the brainstem in blood pressure regulation and hypertension.

## Data and code availability

The UK Biobank resource is open for eligible researchers upon application (http://www.ukbiobank.ac.uk/register-apply/). We used publicly available GWAS data of brainstem^18^ and blood pressure^21^ traits. The GWAS summary statistics for blood pressure traits derived in the present study are made publicly available upon publication in the GWAS Catalog (https://www.ebi.ac.uk/gwas/) and UK Biobank Returns Catalog (https://biobank.ndph.ox.ac.uk/showcase/docs.cgi?id=1).

We made use of publicly available resources for processing the image data and for conducting statistical analyses. We extracted data from individual UK Biobank baskets using the ukb_helper.py script (https://github.com/precimed/ukb). The project R scripts for analyzing brainstem volumes and blood pressure traits are available at https://osf.io/fnxyg/. We performed the genetic overlap analyses with publicly available methods.^22^^,^^24^^,^^25^

## Acknowledgments

This research has been conducted using the UK Biobank Resource under application no. 27412. We performed this work on the Services for Sensitive Data (TSD), University of Oslo, Norway, with resources provided by UNINETT Sigma2, the National Infrastructure for High-Performance Computing and Data Storage in Norway. We obtained funding from the 10.13039/501100005416Research Council of Norway (grant no. 223273, 326813, 324252, 324499, and 323961); the 10.13039/501100006095South-Eastern Norway Regional Health Authority (grant no. 2017112 and 2022080); the 10.13039/501100002347German Federal Ministry of Education and Research (grant no. 01ZX1904A); the European Union’s Horizon2020 Research and Innovation Programme (CoMorMent project; grant no. 847776); and the 10.13039/501100000781European Research Council StG (grant no. 802998).

## Author contributions

*Study design:* T.P.G, T.E., O.A.A. *Manuscript preparation:* T.P.G, T.E., S.B., A.S., O.A.A. *Data interpretation:* T.P.G., T.E., S.B., Z.R., O.A.A. *Statistical analyses:* T.P.G., S.B., Z.R. *Figures:* T.P.G., S.B., Z.R., A.S. *Data preparation and management:* T.P.G., S.B., Z.R., D.E.A.G, D.M., L.T.W., O.A.A. *Genetic insight:* T.E., S.B., Z.R., A.S., O.F., T.K., I.E.S., O.A.A. *Clinical insight:* T.E., S.H., O.A.A. *:* T.P.G., L.T.W., O.A.A. All authors revised the manuscript and approved the final version T.P.G, T.E., O.A.A. *Manuscript preparation:* T.P.G, T.E., S.B., A.S., O.A.A. *Data interpretation:* T.P.G., T.E., S.B., Z.R., O.A.A. *Statistical analyses:* T.P.G., S.B., Z.R. *Figures:* T.P.G., S.B., Z.R., A.S. *Data preparation and management:* T.P.G., S.B., Z.R., D.E.A.G, D.M., L.T.W., O.A.A. *Genetic insight:* T.E., S.B., Z.R., A.S., O.F., T.K., I.E.S., O.A.A. *Clinical insight:* T.E., S.H., O.A.A. *Funding:* T.P.G., L.T.W., O.A.A. All authors revised the manuscript and approved the final version.

## Declaration of interests

O.A.A. has received speaker’s honoraria from Lundbeck, Janssen, Otsuka, and Sunovion, and is a consultant to Cortechs.ai. T.E. has received consultant honoraria from BrainWaveBank and Sunovion and speaker’s honoraria from Lundbeck and Janssen Cilag.

## Supplemental information

Document S1. Figures S1–S13, Tables S1 and S2, and Notes S1 and S2

Data S2. Tables S10–S64

Document S2. Article plus supplemental information
